# An Integrative Approach to Computational Modelling of the Gene Regulatory Network Controlling *Clostridium botulinum* Type A1 Toxin Production

**DOI:** 10.1371/journal.pcbi.1005205

**Published:** 2016-11-17

**Authors:** Adaoha E. C. Ihekwaba, Ivan Mura, John Walshaw, Michael W. Peck, Gary C. Barker

**Affiliations:** 1 Gut Health and Food Safety, Institute of Food Research, Norwich Research Park, Colney, Norwich, United Kingdom; 2 Department of Industrial Engineering, Universidad de los Andes, Bogotá, Colombia; 3 School of Computing Sciences, University of East Anglia, Norwich, United Kingdom; Institute for Systems Biology, UNITED STATES

## Abstract

*Clostridium botulinum* produces botulinum neurotoxins (BoNTs), highly potent substances responsible for botulism. Currently, mathematical models of *C*. *botulinum* growth and toxigenesis are largely aimed at risk assessment and do not include explicit genetic information beyond group level but integrate many component processes, such as signalling, membrane permeability and metabolic activity. In this paper we present a scheme for modelling neurotoxin production in *C*. *botulinum* Group I type A1, based on the integration of diverse information coming from experimental results available in the literature. Experiments show that production of BoNTs depends on the growth-phase and is under the control of positive and negative regulatory elements at the intracellular level. Toxins are released as large protein complexes and are associated with non-toxic components. Here, we systematically review and integrate those regulatory elements previously described in the literature for *C*. *botulinum* Group I type A1 into a population dynamics model, to build the very first computational model of toxin production at the molecular level. We conduct a validation of our model against several items of published experimental data for different wild type and mutant strains of *C*. *botulinum* Group I type A1. The result of this process underscores the potential of mathematical modelling at the cellular level, as a means of creating opportunities in developing new strategies that could be used to prevent botulism; and potentially contribute to improved methods for the production of toxin that is used for therapeutics.

## Author Summary

*Clostridium botulinum* produces botulinum neurotoxins (BoNTs), highly potent substances responsible for botulism. Currently, mathematical models of *C*. *botulinum* growth and toxigenesis are largely aimed at risk assessment and do not include explicit genetic information. In this paper we present modelling based on the integration of diverse information from experimental results available in the literature. Experiments show that production of BoNTs depends on the growth-phase and is under the control of positive and negative regulatory elements at the intracellular level. Here, we integrate these regulatory elements into a combined model of population dynamics and gene regulation to build the first computational model of toxin production at the molecular level. We conduct a validation of our model against several items of published experimental data for different wild type and mutant strains of *C*. *botulinum* Group I type A1. The result of this process underscores the potential of mathematical modelling at the cellular level, as a means of creating opportunities that could be used to prevent botulism, and potentially contribute to improved methods for the production of toxin used for therapeutics.

## Introduction

Commonly found in any soil or water environment, the spore forming Gram-positive rod-shaped bacterium *Clostridium botulinum* and two other clostridia (*C*. *baratii* and *C*. *butyricum*) can, under suitable anaerobic conditions, release botulinum neurotoxins (BoNTs) [1,2]. BoNTs are highly potent substances with an estimated human lethal dose of ~30-100ng [1,2], and are the most powerful toxins affecting human and animal health. BoNTs cause botulism, a severe neuro-paralytic disease that can lead to death in humans as well as in a range of other mammals and birds [3,4]. BoNTs enter into the blood stream in one of three ways: (1) toxin production by bacteria that have colonized the digestive tract of either children less than 12 months of age (infant botulism) or individuals with a suppressed normal intestinal flora (e.g., following antibiotic treatment) including those that have anatomical or functional bowel abnormalities (Adult intestinal toxemia botulism) [2,5,6]; (2) infection and toxin formation in a wound (wound botulism) [5,7,8]; and (3) following oral ingestion of pre-formed toxin in contaminated foods (foodborne botulism) [2,5]. BoNTs target the peripheral motor nerve terminals, blocking neurotransmission by selectively hydrolysing proteins that are involved in the fusion of synaptic vesicles with the presynaptic plasma membrane, thereby preventing acetylcholine release [5,6,9–11].

Foodborne botulism is a severe and sometimes fatal disease [2]. Although there are fewer cases of foodborne illness caused by *C*. *botulinum* than by bacteria of the *Salmonella* genus, the death rate from botulism is relatively high, 17.3 percent, compared with 0.5 percent for *Salmonella* [12]. Thus, the severity of the disease and the widespread presence and persistence of *C*. *botulinum* spores make botulism a global health concern and a cause for vigilance [2].

Seven serotypically distinct botulinum neurotoxins (serotypes A-G) and more than 40 different subtypes [6,9,13] are produced by six phylogenetically distinct clostridia (*C*. *botulinum* Groups I-IV and some strains of *C*. *baratii* and *C*. *butyricum*). Considering the highly potent nature of the neurotoxin, methods that limit the proliferation of *C*. *botulinum* and the associated neurotoxin production in food are a major priority for the food-processing industry; these processes are complicated by the physiological differences among clostridia.

The structures and the mechanisms of action of BoNTs are reasonably well established [11,14–20], however, regulation of botulinum neurotoxin gene (*bont*) expression and BoNT production are not fully understood. Likewise, the environmental signals which trigger the synthesis of the BoNTs and the regulatory network and actors which control the production of the toxin (and many subsequently regulated genes) remain to be elucidated.

What is known, firstly, is that *in vitro* experimental methods developed and applied to monitor *bont* gene expression in *C*. *botulinum* show a peak in neurotoxin gene expression during late exponential and early stationary phase of population growth; expression decreases drastically during stationary phase for *C*. *botulinum* Group I type A [21–26] and for *C*. *botulinum* Group II type E [22,27]. However some of these studies examined a relatively small number of time points during population growth so that the full *bont* gene expression profile is not always observed. Moreover, these studies show that the quantity of BoNT produced can be influenced by the strain, by culture conditions and by the nutritional status of the medium—although the precise mechanisms are unknown.

Secondly, *bont* gene expression is reported to be tightly regulated through positive and negative regulatory systems. Positively, through the participation of BotR [17], Agr quorum sensing system [28], CodY [29] and CLC_1094/CLC_1093 (equivalent to CBO_1042/CBO_1041), CLC_1913/ CLC_1914 (equivalent to CBO_1967/ CBO_1968) and CLC_0663/CLC_0661 (equivalent to CBO_0608/CBO_0607) two component signal transduction systems [30]. Negatively, through the participation of CBO0787/CBO0786 (equivalent to CLC_0843/CLC_0842) [31] which is also a two component signal transduction system [32].

Thirdly, in most *C*. *botulinum* Group I type A1 strains, the genes encoding the neurotoxin (*bont*) and its associated non-toxic neurotoxin proteins (ANTPs) (*ntnh*, *ha*s) are located in a gene cluster and are organized in two transcriptional units (or operons), namely, the *ntnh-bont* and *ha* operons [9,33]. The first operon (*ntnh-bont*), which is located at the 3′ end of the botulinum locus, encompasses the *bont* gene immediately preceded by the *ntnh* gene. Both genes are co-transcribed in the same orientation, and the organization of this operon is highly conserved in all botulinum toxin forming clostridia. The second operon contains the *ha* genes and differs slightly between the various subtypes (BoNT/A1, A5, B, C, D and G). The *ha* operon contains successive genes for the 33 kDa (*ha33*), 17 kDa (*ha17*), and 70 kDa (*ha70*) hemagglutinins [30,34]. These hemagglutinin genes are localised upstream of the *ntnh-bont* genes and are transcribed in the opposite orientation [5,35,36]. Thus, the nontoxic proteins for subtype A1 include NTNHA (which together with BoNT forms the minimally functional progenitor toxin complex (M-PTC)) and three hemagglutinin (HA) proteins (HA70, 17 and 33), which assemble (with the M-PTC) to form the large size toxin complex (L-PTC) [10,37,38].

Lastly, BoNT is released from the bacterium and exists in nature in the form of a complex [36,39,40], i.e. not as a pure toxin [41]. The distinct neurotoxins form complexes of different sizes (from 288 to 900 kDa) by association with ANTPs, i.e. hemagglutinins (HAs) and nontoxic non-hemagglutinins (NTNHs). These ANTPs spontaneously associate with BoNTs at low pH and dissociate at pH 7.5 and above. The associated proteins protect the neurotoxin and facilitate its absorption into the body [37,42].

*C*. *botulinum* Group I type A1 (BoNT/A1) neurotoxins are so far the best characterized neurotoxins, a consequence of both their frequent involvement in human botulism worldwide as well as their greater potency and, therefore, suitability for therapeutics [1].

With all the aforementioned findings, it is reasonable to conclude that *bont* gene transcription and neurotoxin production may be influenced by the bacterial strain. In particular gene transcription may be influenced by the availability of particular nutrients (although the precise mechanisms are unknown) that are present during the transition from late-exponential to early-stationary phase cultures (i.e., growth phase dependent). In turn this transcription is dependent on both positive and negative regulatory elements. This evidence supports the construction of a signal transduction and sensory transcription regulatory network to describe the kinetics of neurotoxin production [32].

Current mathematical models of *C*. *botulinum* are based on statistical data aggregation and describe beliefs concerning the unknown concentrations of *C*. *botulinum* spores in the environment, the uncertain inactivation kinetics for populations of spores at high temperatures and the germination and growth of *C*. *botulinum* populations for a variety of physico-chemical conditions [26,43–51]. These models do not attempt to identify elements of regulatory control which are the key to transferability and to an appreciation of cell to cell variations (in many situations foodborne botulism may be driven by very few cells so that cell variability is a crucial unknown). Furthermore current models sum-up many component processes, such as signalling, permeability and enzymatic activity, obscuring opportunities for improved understanding. The use of computational models amenable to simulation and to the analysis of what-if type scenarios would permit further formulation of hypotheses concerning the gene expression profiles and interactions; additionally a process of iterative computer simulation would guide future experimentation.

In this report we tackle the challenge of integrating the various sources of multi-scale biological evidence into a mechanistic model using ordinary differential equations. In turn this model is used to explain the regulated toxigenesis process of *C*. *botulinum* Group I type A1.

Strains of *C*. *botulinum* Group I type A1 fall into three classes, (i) those that carry the neurotoxin gene in an *ha* cluster, (ii) those that carry the neurotoxin gene in an *orfx* cluster, and (iii) those that also carry a type B neurotoxin gene and form a small amount or no type B neurotoxin. We focus on the first of these, strains that carry the neurotoxin gene in an *ha* cluster [9,52]. We use biological data from the literature that relates to five strains of *C*. *botulinum* Group I type A1 (ATCC19397, 62A, Hall A, Hall A-*hyper* and ATCC3502); the close relationship between these strains having been established by whole genome sequencing, microarray analysis and MLST [53–56]. Additionally, these five strains all possess identical or very similar *bont* and *botR* genes [52,57,58]. This modelling task, to the best of our knowledge, has not been approached so far. We first review the experimental knowledge that has been published in the literature on the patterns of toxin production and toxin gene expression and then identify the main aspects of the regulation, highlighting the key molecular players. The main contribution of this report is shown in the results section where the diverse available information used in constructing the mathematical model of toxin formation by *C*. *botulinum* Group I type A1 is integrated into a complete model in an incremental way. The proposed model is implemented and simulated, to confirm its ability to reproduce the observed patterns of behaviour, in various wild type [WT] strains and in various mutant strains that have previously been experimentally characterized. We further discuss the results and focus on a review of the hypotheses that were made throughout the modelling process, identifying the opportunities they offer for the definition of specific experimental settings that would help in shedding light on several of the poorly understood steps in the process of toxin formation by *C*. *botulinum* Group I type A1.

## Materials and Methods

This study is primarily focused on the mathematical modelling of gene expression, toxin production and population growth that are observed in strains of *C*. *botulinum* Group I type A1. The published data were reviewed to determine which observations could be expressed by a deterministic model. The model was built in an incremental way, using a process which incorporates increasing levels of detail concerning the toxin regulation and production processes, to give a form that could be tested against additional observations.

### Experimental data “input” to the mathematical model

#### Culture conditions and growth parameters for the population dynamics model

Since *bont* gene expression is growth phase dependent, and the concentration of toxin released in the botulinum growth medium is related to the number of bacterial cells, creating a comprehensive computational model of BoNT production, requires growth data expressed in terms of microbial concentration (i.e., reported as viable cell counts (cfu/ml)). For this reason, the experimental growth data reported in [59] and in Figure 7 of [2] were selected because they express viable cell counts (cfu/ml) and take the form of a time-course following the population dynamics.

In these studies *C*. *botulinum* Group I type A1 strain ATCC 19397 (NCTC 7272) was considered. This strain is typical for *C*. *botulinum* Group I type A1 strains and has the *bont* gene in a *ha* neurotoxin gene cluster [9,53]. Strain ATCC 19397 was grown in anaerobic (N_2_/CO_2_/H_2_; 85:5:10) peptone-yeast-glucose-starch (PYGS) medium at 37°C [2,59]). The viable count was determined by plating appropriate dilutions onto VLB agar plates [60] incubated at 30°C for 48hrs under atmosphere of CO_2_/H_2_ (10:90 v/v). The data were used to identify distinct phases of the culture growth which then became the main input to the formation of hypotheses concerning physiological aspects of the bacterium that may determine the observed pattern of growth. In this sense this approach adds to existing empirical models [44].

The reported data in [59] also includes measurements of the quantity of toxin released in the supernatant over time. This was quantified using an endopeptidase activity assay developed by Sesardic and colleagues, and validated against the mouse bioassay [61]. This time series was used for verifying predictions obtained from the models that couple the population dynamics with the toxin production regulation network.

#### Validation through the analysis of additional datasets

Several *in vitro* methods have been developed and applied to monitor expression of the *bont* gene in *C*. *botulinum*, including a gene reporter system, competitive reverse transcription (RT)-PCR, and quantitative RT-PCR [21–24,26]. The kinetics of botulinum toxin gene expression have been investigated in *C*. *botulinum* Group I type A1 strains 62A [21], Hall A [22,23], Hall A-*hyper* [21], ATCC19397 [24] and ATCC3502 [24,26] during the growth cycle. RT-PCR was used to quantify *bont* gene expression, whilst the neurotoxin concentrations in these culture supernatants were measured using an enzyme-linked immunosorbent assay (ELISA). In these studies, growth was measured using optical density (OD) measurements.

### Computational modelling methodology

The proposed mathematical model is based on a continuous-deterministic approach, where the components of the model, such as concentrations, number of bacterial cells etc., are represented as continuous variables and their variation over time is expressed through their first derivative. The dynamics of the multi-component system corresponds with a set of coupled ordinary differential equations for which numerical solutions are obtained by computer simulation. To avoid dealing with the mathematical details of differential equations, we adopted a reaction-based specification language to describe the interactions among the model variables. The whole modelling process is supported by the COPASI modelling and simulation software package [62], which takes as its input the reaction-based specification of the model, and provides the simulated time courses of the variable dynamics. To simplify the process of model definition, we used an incremental procedure which allowed us to build increasingly complex versions of the model, each one incorporating additional pieces of biological evidence and some additional modelling assumptions.

The modelling approach considers two separate levels of representation: (1) at the cell level, the dynamics of the population in a culture and (2) at a sub-cellular level, the network that regulates toxigenesis and gene expression. At a cell level the model describes the dynamics of the consumption of nutrients and of quorum-sensing signals in the culture. This is then coupled with the dynamics of the regulation and gene-expression which is described by the sub-cellular level.

## Results

We first present the population growth sub-model that was previously introduced in [44], and then show how this is coupled to the gene regulation sub-model. The final model resulting from the union of the two sub-models is encoded into COPASI and simulations are used to illustrate the ability to reproduce a selected set of additional experimental results.

### A nutrient and quorum-sensing regulated population growth model

Details of a computational model for the growth of a population of *C*. *botulinum* Group I type A1 cells in a culture have been reported by [44]. As thoroughly explained in [44], the rationale underlying the need of modelling population dynamics is rooted in the experimentally observed correlation between the bacterial growth phase and the toxin production process. Further evidence supporting this correlation at genetic regulation level is described in the section on the molecular model of BoNT synthesis regulation.

The mathematical modelling of *C*. *botulinum* cultures is based on a compartmentalization of the growing population of cells into three distinct groups:

- Adapting cells, denoted by AC, which includes the bacterial cells after their addition to the botulinum growth medium. While the metabolic processes involved remain to be established, they may be similar to that reported in *Salmonella* [63];
- Reproducing cells, denoted by RC, formed by the cells that are actively reproducing;
- Sporulating cells, denoted by SC, which consists of the cells that are committed to sporulation (though not measured in [59]).

**Fig 1:**
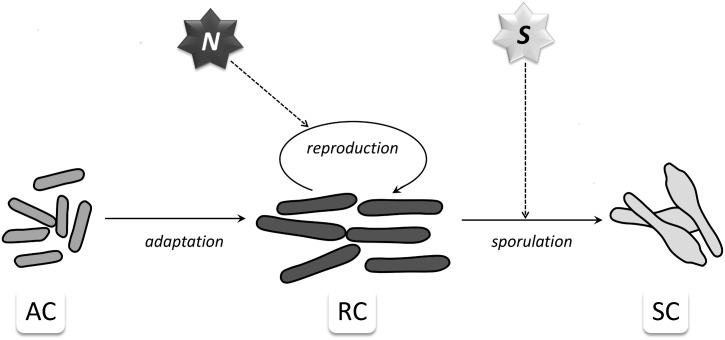
Schematic representation of the population dynamics model. Diagrammatical representation of the best fitting model determined in [44]. The reproduction of cells is controlled by the abundance of nutrients *N*, and the sporulation is regulated by the concentration of a quorum-sensing signal *S*.

The initial population of *C*. *botulinum* cells is fully composed of AC cells, which later evolve to RCs and may commit to sporulation and become SCs. These processes are influenced by some biochemical species generically termed “Signal”, as shown in Fig 1. A future development, not currently included, is to extend the present analysis to start with bacterial spores and to therefore incorporate steps for spore germination and outgrowth [64].

Different hypotheses relating to the nature of the “signal(s)” (previously described in [44]) led to the discrimination of plausible modelling scenarios and were used to generate corresponding models that were then evaluated on their ability to reproduce the observed pattern of growth observed for *C*. *botulinum* type A1 strain ATCC 19397.

We found that a model where two distinct signal sources were considered—the first one determined by the abundance of nutrients essential to *C*. *botulinum* cell growth, which we denoted by the abstract species *N*, and a second one endogenously produced by the bacterial cells and used as a quorum-sensing signal, denoted by *S–*was most successful at explaining the pattern of growth observed for *C*. *botulinum* type A1 strain ATCC 19397. A diagrammatic representation of this modelling option is included in Fig 1. In this model the rate of cell reproduction increases with the nutrient concentration, *N*, whilst the rate of sporulation increases with the concentration of the chemical signal *S*. The model proposed in [44] was encoded using eight reactions. We consider here an updated version, still based on the same rationale, which is encoded by the six reactions listed in Table A of Supporting Information File 1 (Table A in S1 Text). As previously reported in Figure 6 of [44], this model produces a good fit for the experimental growth data generated for strain ATCC 19397.

### Molecular model of BoNT synthesis regulation

**Fig 2:**
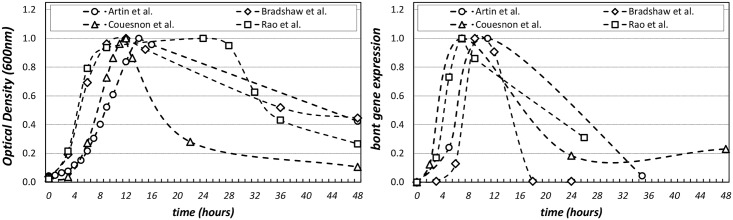
Comparison of population dynamics and *bont* gene expression for *C*. *botulinum* type A1 cultures. Data from the experimental results published in [26], [21], [22] and [23] for *C*. *botulinum* type A1 strains ATCC3502, Hall A-hyper, Hall A and Hall A respectively. Notice that toxin loci of these three strains are genetically identical with each other [9]. Comparison of the time courses measured in optical densities for the cultures (left) and the comparison of the *bont* gene expression time courses (right). Data normalized to the maximum OD (left) and maximum expression level (right) of the single original time course.

Several environmental stimuli have been identified with positive and negative regulation of toxin production in *C*. *botulinum* Group I type A1. Neurotoxin production has been reported to be associated with the transition from late-exponential to early-stationary phase cultures. This is indicated by a peak in the level of neurotoxin gene cluster expression that is clearly observable in the late-exponential to early-stationary phase of cultures and which drastically decreases during the later stationary phase (as shown in Fig 2). Moreover, the expression patterns for all the genes, in both the *ntnh/bont* and the *ha* operon, show an equivalent correlation with population dynamics (data available in [26], [21], [22] and [23]). This points to regulatory elements that link population growth to toxigenesis in *C*. *botulinum* type A1.

#### BotR as a positive regulator of BoNT synthesis

Botulinum neurotoxins are produced in the form of a complex containing the neurotoxin itself and one or more non-toxic auxiliary proteins that protect the neurotoxin from environmental stress and assist in absorption [65]. A majority of type A1 toxins are complexed with the non-toxic non-hemagglutinating (NTNH) protein and three hemagglutinins (HA17, HA33 and HA70) [26,40]. The genes coding for these proteins are organized in two operons, namely the *ntnh-bont* and *ha* operons [21], and the *botR* gene can be found between the two.

The *botR* gene encodes a 21-22kDa protein (BotR), an alternative sigma factor with features of a DNA-binding protein (i.e., highly basic isoelectric point and helix-turn-helix motif [17]). BotR appears as a key positive regulator for the *ntnh-bont* and *ha* operons. Indeed, both operons have consensus -10 and –35 core promoter sequences, which are recognized by BotR, which specifically binds to the promoter region of the *ntnh-bont* and *ha* operons and directs RNA polymerase (RNAP) to transcribe the two operons [66]. The *botR* gene is transcribed in the same orientation as *bont*, and BotR has been characterised as a transcriptional activator of *ntnh-bont* and *ha* genes based on *botR* overexpression or partial inhibition by antisense mRNA in *C*. *botulinum* Group I type A1 [17,30,66]. BotR can also target its own promoter, but initiation of transcription could not be observed *in vitro* [67].

Based on this evidence, BotR is included in the coupled model as a direct positive regulator of toxigenesis as well as a positive regulator of itself.

#### TCSs as positive and negative regulators of BoNT synthesis

Experiments reported by [30] focused on the toxin regulatory elements in the genome of *C*. *botulinum* Group I type A1 strain Hall. In this study, the authors first identified a considerable number (30 in total) of gene pairs coding for two-component systems (TCSs) that affected toxin regulation. TCSs are widely used in bacterial stimulus-response coupling for sensing and relaying a variety of environmental and developmental cues that affect gene activation. A TCS consists of a membrane-bound histidine kinase, that senses a specific stimulus, and a response regulator that typically has the characteristics of a DNA binding protein to mediate the expression of a set of target genes [68]. The signal is relayed from the sensor component to the response regulator via trans-phosphorylation. The role of the TCS candidates were explored by [30] using antisense mRNA silencing to determine which were primarily acting on toxin operons. The search led to the identification of three TCSs that were shown to positively regulate toxin production. These results indicate (please note we will use the CBO equivalent numbers identified for strain ATCC3502):

1 The three TCSs which positively regulate toxin production, are encoded by the gene pairs *cbo_1042/cbo_1041*, *cbo_1967/cbo_1968*, and *cbo_0608/cbo_0607*;
2 The effects of the three TCSs are independent from that of BotR, since expression of *botR* is not significantly affected by the mRNA silencing;
3 The CBO0608/CBO0607 TCS was suggested to be homologous to TCSs of the PhoP/PhoR family involved in, but not restricted to, sensing and reacting to phosphate starvation.
  These experimental results led us to include two distinct positive regulatory mechanisms in our model: a first one that models the effect of the CBO0608/CBO0607 TCS, which we assume is sensing and reacting to the lack of nutrients, and a second one (consisting of the two species CBO_SHK/CBO_RR) that abstractly represents the two TCSs CBO_1042/CBO_1041 and CBO_1967/CBO_1968, which we assumed to be activated by the increase in concentration of quorum-sensing molecules.

Furthermore, the first reported evidence of negative regulation of *C*. *botulinum* Group I type A1 toxin synthesis was provided by Zhang *et al*. [31], who showed that the CBO_0787/CBO_0786 TCS down-regulates toxin production in strain ATCC 3502. The experimental results [31] most relevant to the coupled model are:

4 Expression of the TCS components CBO_0787 and CBO_0786 is dependent on the growth phase with a constant level of expression preceding entry to the late exponential phase followed by a subsequent reduction of about 80 percent;
5 The *cbo0787* and *cbo0786* genes are transcribed polycistronically;
6 Phosphorylated CBO_0786 negatively regulates toxin production, by binding directly to the conserved -10 site of the core promoter regions of *ntnh-bont* and *ha* operons so blocking BotR-directed transcription.

Based on this experimental evidence we can infer, and include in the coupled model, a role for the CBO_0787/CBO_0786 TCS as a direct negative regulator of toxigenesis, with phosphorylated CBO_0786 acting as the species exerting the repression by direct binding to the toxin gene promoters.

#### Nutrition-related metabolic and quorum-sensing pathways as regulators of BoNT

So far, we have identified elements for the model construction without identifying the specific mechanisms for coupling i.e. initiation of response. Experimental evidence indicates that botulinum neurotoxin production is affected by the availability of various carbon and nitrogen sources. Multiple research works [21–23,26,59] have quantified the effect that nutrients have on the toxin production. Nutrient(s) availability is already included in the population dynamics element of the coupled model and additionally we hypothesize that the abundance of nutrient(s) also regulates toxigenesis directly.

A recent report by Zhang and colleagues [29] demonstrated the role of the global regulator protein CodY in toxin synthesis and elements of this observation provide support for a plausible picture of the nutrition-related effects on toxigenesis in *C*. *botulinum* Group I type A1:

1. CodY is able to bind to the promoter region of the *ntnh/bont* operon;
2. The binding affinity of CodY for the promoter regions of *ntnh/bont* operon increases in GTP rich conditions;
3. *codY* mutant strains show reduced expression levels of *bont* (approximately 50% less) compared to wild type;
4. The temporal pattern of expression of *bont* is the same in *codY* mutant strains and wild type;
5. Two putative binding regions, each one with three mismatches to the consensus CodY-binding motif, are found upstream of the CBO_0787/CBO_0786 operon.

Points 3 to 5 imply that the overall role of CodY is to activate toxin production. However, points 1 and 2 both imply that the effect of CodY is maximal on the operon when the availability of nutrients is high, i.e., when no toxin is produced. Therefore, the binding of CodY to the promoters of *ntnh/bont* operon must be exerting a repression effect on transcription. That is to say, the activation effect of CodY must be the result of an additional regulation exerted by CodY. Together this means that CodY may be repressing the repressor TCS CBO_0787/CBO_0786 by directly binding to the TCS promoter. For this reason, in a CodY mutant the repression effect of CBO_0787/CBO_0786 would not be released and the expression of the toxin genes is reduced. Point 4 of the sub-section on TCSs as positive and negative regulators of BoNT synthesis indicates that this repression effect of CodY needs to be exerted after the late exponential phase.

We have conducted a sequence analysis of the *botR* promoter region and found an additional putative binding region for CodY, with some noticeable similarities to sequence motifs and an associated CodY-binding sequence previously identified in the CodY-regulated promoter of another *C*. *botulinum* ATCC 3502 gene [69]. This is therefore consistent with the hypothesis that CodY regulates the expression of the alternative sigma factor BotR (see also Supporting Information File 3 for details (S3 Text)). Since we know that *botR* expression is also phase dependent, we make the assumption that CodY regulates BotR positively, so that when the available nutrient(s) decreases, CodY begins to exert an activation effect on the *botR* gene transcription. As a consequence, we suppose in our modelling that CodY regulates toxigenesis via two routes, activation and repression, in distinct phases of the population growth.

For modelling, we assume the existence of two distinct forms/behaviours of CodY; one we named CodY1, which is prevalent when available nutrient(s) is high, and the other we named CodY2, which accumulates when nutrient(s) are scarce. The transition between the two forms is regulated by the quantity of nutrient(s). In the model, CodY1 represses the *ntnh/bont* operon, while CodY2 represses the CBO_0787/CBO_0786 operon and upregulates the *botR* gene transcription. We do not model the mechanism underlying the proposed two behaviours of CodY, but this could involve presence/absence of a bound cofactor or interactions with, or recruitment of different activator/repressor components.

The CBO_0787/CBO_0786 TCS, which has expression regulated by CodY2, is activated via phosphorylation of the CBO_0787 histidine kinase in response to an unknown signal. We assume in the model that the signal is indirectly relayed by the nutrient(s), and therefore by modelled species *N*. Moreover, since the CBO0608/CBO0607 TCS is assumed to be involved in, but not restricted to, sensing and reacting to phosphate starvation [30], we have placed its regulation under the control of the nutrient(s), by assuming that the phosphorylation of the CBO0608 histidine kinase is repressed by modelled species *N*.

The molecular details of the quorum-sensing pathway regulating toxin production in *C*. *botulinum* Group I type A1 strains has not yet been clarified. What is known from the work reported in [28] is that the genome includes two regions, *agrD1* and *agrD*, which code for homologues of the *Staphylococcus aureus* agr-like quorum sensing system. Moreover, the authors [28] demonstrated that in the closely related organism *C*. *sporogenes*, the pattern of expression of the genes in corresponding regions is strongly correlated with the growth phase: i.e., it increases throughout exponential growth, peaking at late exponential phase, and considerably drops once stationary phase is reached. The authors showed that, the insertional inactivation of the genes in the *agrD1* and *agrD2* regions in *C*. *botulinum* Group I type A1 (strain ATCC 3502) resulted in a reduction in the amounts of toxin produced. More precisely, inactivation of agrD1 led to a marked reduction of the early toxin production, with a return to wild-type levels during late-stationary phase, whereas inactivation of agrD1 led to a more severe restriction of the toxin production that persists throughout the population growth.

Although this experimental evidence clearly indicates a role for quorum-sensing in toxigenesis, the available information is not sufficient to make hypotheses about possible modelling options relating to the pathways that link quorum-sensing with gene expression. We however, decided to include the action of quorum-sensing into the gene expression sub-model in an abstract way. We make a hypothesis that the TCSs CBO_1042/CBO_1041 and CBO_1967/CBO_1968 shown to regulate toxin synthesis in a positive way, sense and react to changes in concentration of a quorum-sensing *signal*, represented as modelled species *S* in the population sub-model of the section on nutrient and quorum-sensing regulated population growth model.

#### Computational model

In the integrated model we include the known regulatory mechanisms controlling toxin production, but not the details of the toxin assembly nor secretion, nor other processes yet to be fully deciphered. Moreover, we limit the model scope to neurotoxin synthesis (i.e., BoNT protein), not as a complex (without the associate proteins, NTNH the HAs, and their interactions). Even though, these simplifications were made in order to prevent the introduction of a large number of unknown kinetic parameters, it is important to note that NTNH, which is transcribed polycistronically from the *ntnh-bont* operon, is subject to the same regulation as BoNT. As for the *ha* operon, it is also transcriptionally regulated by BotR, as well as by the three positive regulatory TCSs as shown in [30], and the negative regulatory TCS, as shown in [31]. Thus, we assume that the ANTPs would exhibit the same pattern of expression as BoNT.

The integrated model includes BoNT synthesis and export as a single process, and assumes a delay in export to the culture supernatant.

The model includes the transcription of each species for which the synthesis process is known to be regulated, i.e., the CBO_0787/CBO_0786 proteins, the alternative sigma factor BotR and the *bont* gene. For these species, transcription and translation are modelled altogether, to avoid introducing too many unknown kinetic parameters into the model. All the synthesis processes are regulated by the abundance of nutrient(s) (modelled species *N*). As there is no available information on the regulation of the expression for the proteins of the TCSs, CBO_1042/CBO_1041 and CBO_1967/ CBO_1968, we do not include their synthesis processes in the model. Instead we assume a constant concentration of the constituent proteins which change between their unphosphorylated and phosphorylated forms depending on the abundance of regulators. Similarly the model does not include the synthesis process for CodY.

**Fig 3:**
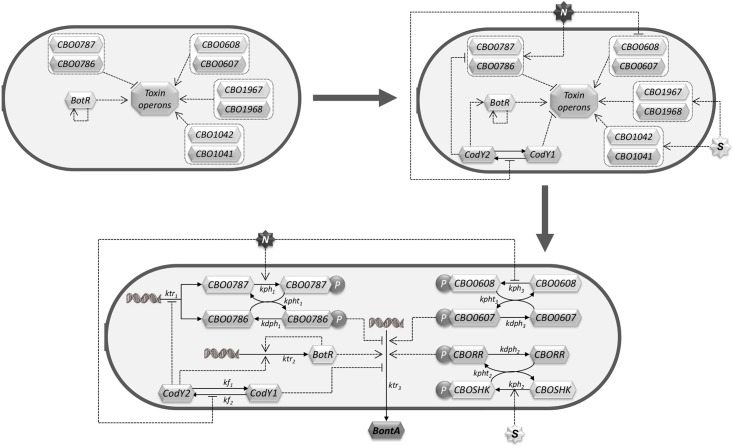
Diagrammatic representation of the computational gene expression sub-model. (Top Left) show the role of the BotR sigma factor, and of the three TCSs reported to regulate positively toxigenesis in *C*. *botulinum* Group I type A1 strain, along with the negative TCS regulator. (Top Right) our hypothesis of how the availability of nutrients (species N) regulates directly and indirectly (via CodY) toxin production, and how the quorum sensing signal (species S) together with the two positive TCS regulators, recognise the quorum-sensing pathway whose effect on toxin production was experimentally observed in the work of Cooksely and colleagues [28]. (Lower), the dashed arrows represent regulation mechanisms, whereas solid lines model mass transfer reactions. The species N (Nutrients) and S (quorum-sensing signal) are shared with the population sub-model. The state of each bacterial cell is assumed to be the same. Species CBO_0786, CBO_0787 (and their phospho forms), BotR and BoNT are subject to degradation (reactions not graphically depicted).

Finally, we include in the integrated model a degradation reaction for each species synthesised, i.e. for CBO_0786, CBO_0787 (and their phospho forms), BotR and BoNT. The integrated computational model for the gene expression network that regulates BoNT production is illustrated in Fig 3. For the sake of clarity the degradation reactions are not depicted. The gene expression model represents the molecular machinery that regulates toxigenesis inside each bacterial cell. The inner part of the cell is enclosed in the rod-shaped form in Fig 3, and the *N* (Nutrients) and *S* (quorum-signal) modelled species are shared with the population sub-model. We use the same notation of dashed and solid lines as before to distinguish between regulation and mass transfer reactions.

To complete the definition of the model it is necessary to specify, in terms of molecular interactions, the repression and activation effects on the synthesis processes of the negative regulatory CBO0787/CBO0786 TCS and the alternative sigma factor BotR; as well as the impact on the *ntnh/bont* operon.

**Fig 4:**
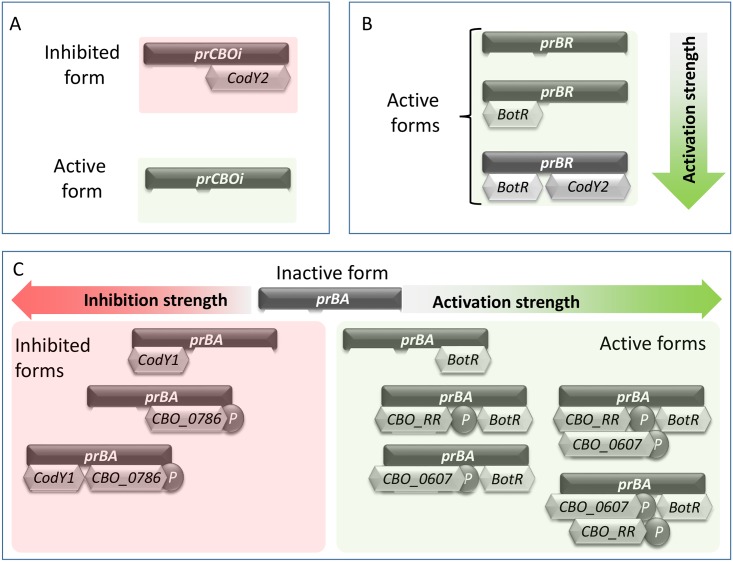
States of the CBO0787/CBO0786, BotR and BoNT promoters. The synthesis of the negative regulatory TCS, of the alternative sigma factor BotR and the BoNT protein are regulated by inhibitory and activator species. (**A**) shows the two possible states of *prCBOi*, the promoter for the polycistronic transcription of proteins CBO0787/CBO0786; (**B**) illustrates the three possible active states of *prBR*, the promoter of BotR; (**C**) details the possible states of the *ntnh-bont* operon promoter *prBA*: inactive, when not bound, inhibited by CodY1 and/or phosphorylated CBO_0786 inhibits transcription, and activated, by BotR and subsequently by phosphorylated CBO_RR and/or phosphorylated CBO_0607 for increasing levels of activation.

We approach this modelling task by explicitly representing as variables of the model the state of the promoters. The promoter of the negative regulatory TCS (named *prCBOi*) is assumed to be in one of two states: inhibited by the CodY2 species, or active, as illustrated in Fig 4A. The promoter of BotR, called *prBR* in the model, has three different states of activation: an initial state, (which can express a basal level of synthesis where *prBR* is not activated by any transcription factor), a second state in which BotR is bound to *prBR* and acts as a self-activator, and a third state in which CodY2 binds *to prBR* next to the already bound BotR. In modelling the promoter activity, we assumed that positive regulatory proteins, i.e. CodY2 and the active forms of CBO_0607 and CBO_RR, act as co-factors in transcription, increasing the stability of the transcription machinery and therefore the synthesis rate. Fig 4B illustrates the different levels of activation of the *prBR* promoter, each one associated with a distinct rate of synthesis.

The activity of the promoters of the *ntnh/bont* operon (*prBA*) is modelled in a similar way but the multiple positive and negative regulators that affect BoNT synthesis give rise to many more states, as shown in Fig 4C. *prBA* is modelled as being inactive, i.e. unable to initiate synthesis, if a positive regulator is not bound to it. That is, if the negative regulatory species *CodY1* and phosphorylated CBO0786 bind to *prBA*, synthesis is inhibited (left complex forms illustrated in Fig 4C). The model also assumes that phosphorylated CBO0786 is a stronger inhibitor than *CodY1*, and that the inhibition strength is maximum when both inhibitors are bound to *prBA*. The active forms of the *prBA* are shown in the right part of Fig 4C. Here, we assume that *prBA* can be activated in three ways: by the binding of BotR on its own, by the combined binding of BotR, a phosphorylated CBO_RR and phosphorylated CBO_0607 or by the simultaneous binding of BotR and both phosphorylated CBO_RR and phosphorylated CBO_0607, with largest complexes being more active than small complexes. The rationale underlying this modelling is that phosphorylated CBO_RR and phosphorylated CBO_0607 play the role of co-transcription factors, stabilising the transcription machinery and increasing the transcription rate of the *prBA* which also requires the alternative sigma factor BotR for transcription initiation.

The overall gene expression model corresponds to a set of 49 reactions which are listed in Table B of Supporting Information File 1 (Table B in S1 Text). The initial state of the whole model, as well as the details of the kinetic rates of the gene regulation network and the population sub-models, is provided in Supporting Information File 2 (S2 Text).

### The computational model is able to reproduce additional experimental results

In this section we expound the procedure used for calibrating model parameters, and then proceed to validate the model by checking against characteristics of toxigenesis which have been reported previously in sections on nutrient and quorum-sensing regulated population growth model and the molecular model of BoNT synthesis regulation.

To find suitable values for model parameters, we used the experimental data from [59] for type A1 strain ATCC 19397, which we considered as the 'wild type' organism for the purpose of our modelling (WT, hereafter). The experimental time course for the population size (measured in CFU/ml over time in [59]) provides the parameters of the population sub-model, i.e. the kinetic parameters of reactions (1) to (6) provided in Table A of Supporting Information File 1 (Table A in S1 Text). The amount of toxin in the supernatant measured in the same experiment in [59] (measured in MLD_50_/ml over time) provides the data for fitting the gene expression sub-model, i.e. the kinetics of reactions (1) to (49) listed in Table B of Supporting Information File 1 (Table B in S1 Text). The model parameters are reported in Supporting Information File 2 (S2 Text).

**Fig 5:**
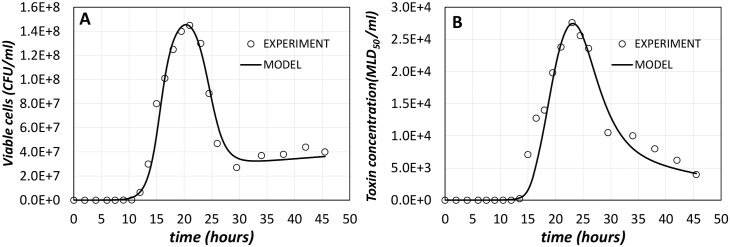
Comparison of experimental data (from [59]) and model predicted results for WT. (A) shows the population dynamics, where data measurements are in CFU/ml over time, while (B) illustrates the amount of toxin in the supernatant. In both plots, experimental data points are drawn as circles, while model predicted data are shown as continuous lines.

The fitted model is compared with the WT experimental data in Fig 5A and 5B. The experimental data points are shown as empty circles, whereas the computational model is reported as continuous lines. There is an inevitable match between model outcomes and the experimental 'model training' data, which is confirmed by analysis of correlation. For the population an R^2^ measure is 0.975 while for the toxin production it is 0.95.

After tuning model parameters to fit WT observed behaviour, we proceeded to validate the model, by assessing its ability to reproduce the behaviours experimentally observed in the different *C*. *botulinum* mutant strains we had considered in the study. We examined four different mutations, which are implemented in the WT model exclusively by changing the initial state of the model, i.e. without any change to the kinetics of the reactions. The mutants we considered for the purposes of our validation are as follows:

- The *cbo0786* mutant constructed by insertional inactivation in Zhang *et al*., 2013 [31], is denoted as C786_M model, and addressed by setting the initial value of the *prCBOi* variable to zero.
- The *codY* mutant constructed by insertional inactivation in Zhang *et al*., 2014 [29], is denoted as CODY_M model, and addressed by setting the initial values of the CodY1 and CodY2 variables to zero.
- The Hall/707 and Hall/714 mutants, constructed by the insertion of DNA anti-sense mRNA strains for the two positive regulatory TCSs CBO_1042/CBO_1041 and CBO_1967/ CBO_1968 in Connan *et al*., 2012 [30], denoted as RR_M model, and addressed by setting the initial value of the CRR variable to zero.
- The Hall/1146 mutant, constructed by the insertion of DNA anti-sense mRNA strains for the positive regulatory TCS CBO_0608/CBO_0607 in Connan *et al*., 2012 [30], is denoted as C607_M model, and addressed by setting the initial value assigned to the CBO_0607 variable to zero.

For each mutant, we obtain and report the toxigenesis predictions (pattern and amount of BoNT) from both the WT model and the mutant model. Then we examine the relationship between model predictions and the experimental results to determine the ability of the models to reproduce wet-lab evidence.

#### Comparison with cbo0786 mutant

We summarize in Table 1 the results reported in Fig 4 (upper panel) and 5A of Zhang *et al*., 2013 [31] for *C*. *botulinum* strain ATCC 3502, which we call wild-type (*wt*), and for the *cbo0786* mutant, which we call *mut*. In the experiments of Zhang *et al*., the relative expression of the *bont* gene and the amount of neurotoxin in the supernatant are quantified at three time points: mid-exponential growth phase (ME, approx. 4 hours), late-exponential growth (LE, approx. 7 hours) and at early-stationary phase (ES, approx. 10 hours).

**Table 1:** Experimental data from Zhang *et al*., 2013 [31]. Data for *bont* gene expression and supernatant toxin concentration of *C*. *botulinum* ATCC 3502 (*wt*) and *cbo0786* mutant (*mut)*, measured at mid-exponential (ME), late-exponential (LE) and early-stationary (ES) phases.

	Relative expression of *bont* (ELISA, normalized to 16S rn)		Neurotoxin in supernatant (OD at 405 nm)	
	*wt*	*mut*	*wt*	*mut*
**ME**	0.85	1.60	0.65	0.88
**LE**	2.60	7.70	0.38	0.90
**ES**	2.10	4.50	0.39	1.50

**Fig 6:**
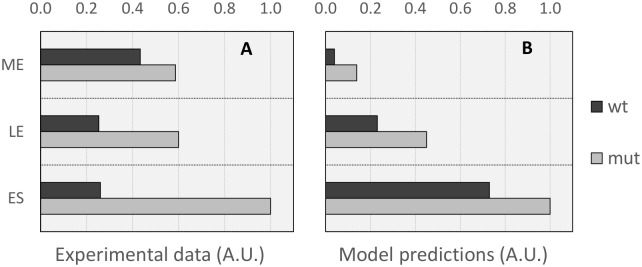
Comparison of experimental and model predictions for concentration of toxin in the supernatant for wild-type and the *cbo0786* mutant studied in [31]. (A): normalized concentration of toxin in the supernatant for *C*. *botulinum* ATCC 3502 (*wt*) and the *cbo0786* mutant (*mut*) as reported in [31] (B): model prediction for toxin concentration in the supernatant (normalized) for *wt* and for the C786_M mutant (*mut*).

We compare our models predictions with the experimental results by showing, in Fig 6A, a graphical representation of the data collected by Zhang *et al*., 2013 [31] for the amount of toxin in the supernatant (i.e. data in the right columns of Table 1) and in Fig 6B the equivalent measures as obtained from our models predictions.

To make the Zhang *et al*. data (which reports concentrations as A at 405 nm) comparable to our model results (which predicts concentrations as MLD50/ml), we normalized both the *wt* and *mut* data to the maximal measured toxin concentration, which in both Fig 6A and 6B corresponds to the amount of toxin measured at data-point ES for *mut*. Also, we defined the ME, LE and ES time points for the model simulated cultures to be 14, 16 and 18 hours, respectively.

Comparing Fig 6A with Fig 6B indicates that the experimentally measured and the modelled *wt* are quite different in terms of the pattern of toxin production. For *wt* the experimental peak of neurotoxin concentration appears in the culture at the ME measurement time; a behaviour that is remarkably distinct from that of the *C*. *botulinum* strain ATCC 19397 we considered as the basis of our modelling in this work. Our model is however able to reproduce the increase in toxigenesis induced by the silencing of the *cbo0786* gene. Indeed, as can be appreciated from Fig 6B, the model of the mutant (mut) consistently produces higher amounts of toxin in the supernatant.

#### Comparison with codY mutant

Zhang *et al*., 2014 [29] measured the amount of toxin in the supernatant in a culture of *C*. *botulinum* strain ATCC 3502, which we will consider as wild-type (*wt*) in this section, and for a *codY* mutant constructed by insertional inactivation (*mut*, in this section). Data for the measured concentration of toxin in the supernatant of the cultures of *wt* and *mut* as a function of time are summarized in Table 2. These data have been extracted from Figure 3, page 7654 of [29].

**Table 2:** Experimental data from Zhang *et al*., 2014, [29]. Data for the supernatant toxin concentration of *C*. *botulinum* ATCC 3502 (*wt*) and *codY* mutant (*mut)*, measured in μg/ml at various time points during the culture growth.

		Time (hours)						
		5	6	9	12	24	48	96
**Toxin concentration in supernatant (μg/ml)**	***wt***	0.12	0.22	0.38	6.95	41.5	60.5	50.5
	***mut***	0.06	0.11	0.14	2.95	17.5	30.5	29.5

**Fig 7:**
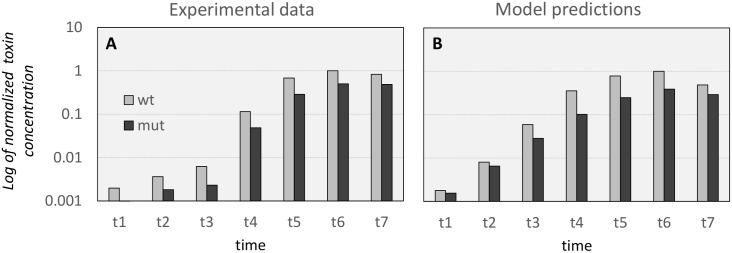
Comparison of experimental results and model predictions for concentration of toxin in the supernatant for the wild-type and the *codY* mutant studied in [29]. (A): normalized observed concentration of toxin in the supernatant for *C*. *botulinum* ATCC 3502 (*wt*) and the *codY* mutant (*mut*), as reported in [29]. (B): model prediction for toxin concentration in the supernatant (normalized) for *wt* and for the CODY_M mutant (*mut*).

To compare our model results with the experimental data reported in Table 2, we defined a sequence of time points that would match the culture growth phase observation times of Zhang *et al*., 2014 [29]. In their report the peak of neurotoxin concentration in the *wt* culture is achieved at time 48 hours and the transition between late exponential and early stationary growth phases occurs at time 9 hours. Therefore, we define the observation time points for the modelled cultures to match those distinctive events (time 17.5 hours for the transition from late-exponential to early-stationary phases, and time 22 hours for the peak of toxin concentration in the supernatant) and we show the comparison between the experimental data and the models predictions in Fig 7. To facilitate the comparison, we denote the two sequences of time points as t1,t2,…t7. Fig 7A shows the log of the toxin concentration in the supernatant, as obtained in the experimental work of Zhang *et al*., 2014 [29], and Fig 7B shows the analogous results obtained from our models. As can be observed, there is a good agreement between experiments and model predictions (particularly the relative values for wild type and mutant).

#### Comparison with Hall/707, Hall/714 and Hall/1146 mutants

Connan and co-authors report in [30] the results of experimental work investigating the role of various two-component systems in toxigenesis regulation. They compare the amount of toxin in the supernatant of a *C*. *botulinum* type A Hall strain culture against the toxin in the supernatant for different mutants in which the two-component systems have been silenced. Of interest for our purposes are the *Hall/707* and *Hall/714* mutants, for which we have built a model named CRR_M, and the *Hall/1146* mutant, for which we constructed a model called C607_M. Since the *Hall/707* and *Hall/714* mutants provide practically identical results in terms of the amount of toxin produced in the supernatant, we only consider Hall/707 in the following. We denote by wild-type (*wt*) the original *C*. *botulinum* type A Hall strain, and by *CRR_M* and *C607_M* the two mutant strains *Hall/707* and *Hall/1146*.

In [30], on page 8, Figure 3D, the authors reported the measured amounts of toxin concentration in the supernatant (A at 405nm), for 3 different time points at 8 hours; which corresponds to a point in the exponential growth phase, at 12 hours, in the early stationary phase, and at 24 hours, well inside the stationary phase.

**Fig 8:**
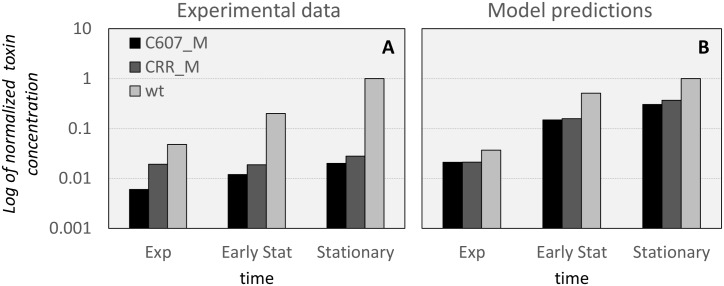
Comparison of experimental results and model predictions for the concentration of toxin in the supernatant for the wild-type (*wt*) and two mutants studied in [30]. (A), experimentally measured amounts of toxin concentration in the supernatant, normalized by the maximal measured concentration (for *wt*, in the stationary phase) and reported on a log scale. (B), predicted toxin concentrations from our *wt*, CRR_M and C607_M mutant models, normalized by the maximal predicted concentration (for *wt*, in the stationary phase), log scale on the vertical axis.

To compare the model predictions with the experimental data reported in Table 3, we choose three time points in the predicted time courses of the toxin supernatant concentration: time 14.5 hours for the exponential growth phase, time 18.5 hours for the stationary phase and time 24 hours for the stationary phase. In Fig 8 we show the amounts of toxin in the supernatant (normalized with respect to the maximum amount, which in all cases corresponds with data for *wt* in the stationary phase) coming from the experiments in Connan *et*. *al* [30] (Fig 8A) and from our model predictions (Fig 8B). It can be seen that the models can reproduce the reduced toxigenesis of both mutant phenotypes and can also identify that the C607_M mutant (i.e. the *Hall/1146* strain) exhibits a larger reduction in toxin concentration.

**Table 3:** Experimental data from Connan *et*. *al* [30]. Data for supernatant toxin concentration of *C*. *botulinum* type A Hall (*wt*) and the mutants Hall/707 (*CRR_M*) and Hall/1146 (*C607_M)*, measured during the Exponential growth phase (time 8 hours), the early stationary phase (time 12 hours) and the stationary phase (time 24 hours).

	Neurotoxin in supernatant(A at 405 nm)		
	*wt*	*CRR_M*	*C607_M*
**Exponential**	12	4.8	1.5
**Early stationary**	50	4.7	3
**Stationary**	250	7	5

## Discussion and Conclusions

For the first time we have defined and implemented a computational model, at the molecular level, for the highly regulated process of BoNT production in *C*. *botulinum* Group 1 type A1.

In contrast to existing modelling approaches, largely aimed at risk assessment for *C*. *botulinum*, this development does not integrate out component processes such as signalling, membrane permeability and metabolic activity, and it does include elements of genetic in-formation. The model captures causal relations among the known regulators of toxigenesis, at the molecular level. This leads to a computational model which is able to embrace both the population dynamics of the cells (so that we were able to include growth phase-dependent patterns of bacterial behaviour) as well as behaviour of the genetic regulatory network and the molecular interactions that link toxin expression with the environmental and population generated signals. The model construction has integrated the available experimental knowledge on the factors that, at a molecular level, regulate toxigenesis in *C*. *botulinum* Group 1 type A1; previously reviewed in [32]. In addition it portrays the effects of nutrient availability and quorum-sensing molecules and their coupling with distinct sensing and response TCSs that are regulators that mediate the activation of the toxin coding genes. The model satisfies a validation based on its ability to predict the effects of various mutations that have been experimentally studied in vitro. The validation results suggest that the model is able to provide a plausible explanation for the interplay of the multiple regulation mechanisms that impact toxin production in C. botulinum Group 1 type A1.

**Fig 9:**
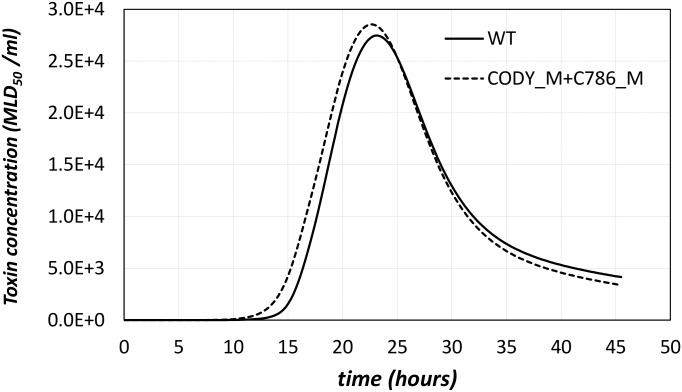
Comparison of predicted levels of toxin supernatant concentration, as obtained from the models of WT (continuous line) and of the double mutant strain CODY_M+C786_M (dashed line). Our model produces a testable prediction for the phenotype of this mutant, which should be similar to WT as far as toxigenesis is concerned.

Models that encode causality have significant advantages over purely statistical descriptions, because they lend themselves to exploration of what-if-scenarios and generating test-able hypotheses. For instance, the model proposed here can be used to predict the pheno-types of mutants that have not yet been studied in vitro. As an example, we can explore the predicted toxin production of a mutant cell where the positive regulator CodY is removed and also the negative regulator TCS CBO0787/CBO0786 is silenced (the CO-DY_M+C786_M double mutant, based on the abbreviation used in the section on the computational model ability to reproduce additional experimental results. We can then compare the predicted concentration of toxin in the supernatant for this double mutant with that obtained by the *C*. *botulinum* WT model (Fig 9). Our model predicts that silencing the CBO0787/CBO0786 TCS rescues the CODY_M mutant ability to produce toxin, to levels similar to those of the wild type. This is a prediction that can be tested in vitro, to provide either further support for the model structure, or to generate new evidence that can be integrated into parameterization and hence improve the predictive capability.

Furthermore, models can be tested for conditions not yet considered in the laboratory set-ting; thus obtaining additional predictions that could be conductive to the definition of experimental settings.

This novel model is an initial attempt to elucidate toxigenesis in *C*. *botulinum* Group 1 type A1. We expect it will require further tuning, improvements and changes. We made a substantial number of assumptions about the dynamics of the activation of promoters, which require experimental confirmation. The process of toxigenesis has been simplified not to include too many unknown details of the hemagglutinins and NTNH synthesis, together with the complexation process that generates the functional forms of the toxin. Clearly it is essential that the amount and quality of experimental results is increased. In the absence of large datasets on a specific genotype, we had to construct the model from experimental data obtained from varying, though closely related, strains. Each study used a different granularity for data collection and a distinct measurement technique, which gave us the challenging task of validating a quantitative model with qualitative data. Continuing with improving the reliability of model predictions and refining the model with the inclusion of additional experimental evidence is the subject of our on-going research work.

## Supporting Information

S1 Text Reactions defining the population sub-model and the gene expression sub-models. (DOCX)

S2 Text Parameters and kinetic rates of sub-models. (DOCX)

S3 Text This supporting text provides further details about the sequence analysis of the *botR* promoter region. (DOCX)
