# Supplementary material for: An Integrative Approach to Computational Modelling of the Gene Regulatory Network Controlling Clostridium botulinum Type A1 Toxin Production
Source: PLoS Comput Biol. 2016 Nov 17;12(11):e1005205. doi: 10.1371/journal.pcbi.1005205 (PMC5113860; doi:10.1371/journal.pcbi.1005205)
Supplement: S1 Text — (DOCX) [file pcbi.1005205.s001.docx]

Supporting Information File 1 – Reactions of the computational model

Table S1. 1: Reactions defining the population sub-model.

| **ID** | **Reaction** | **Description** | **Type of kinetic rate** |
| --- | --- | --- | --- |
| **(1)** | $AC+N\underset{\to}{k_{1}}RC$ | Adaptation of the cells of the culture (lag phase), consumes nutrients | Hill function, regulated by N |
| **(2)** | $RC+N\underset{\to}{k_{2}}RC+RC$ | Reproduction of cells, consumes nutrients, also dependent on signal concentration | Hill function, regulated by N and S |
| **(3)** | $RC\underset{\to}{k_{3}}SC$ | Commitment to sporulation, dependent on signal concentration | Hill function, regulated by S |
| **(4)** | $RC\underset{\to}{k_{6}}RC+S$ | RC cells produce and release the quorum-sensing signal | Mass action |
| **(5)** | $SC\underset{\to}{k_{7}}\alpha\cdot S$ | Sporulating cells expels the quorum-sensing signal at the time of cell lysis. | Mass action |
| **(6)** | $S\underset{\to}{k_{8}}\emptyset$ | Degradation of the signal | Mass action |

Table S1 2: Reactions defining the gene expression sub-model.

| ***ID*** | ***Reaction*** | ***Description*** | ***Type of kinetic rate*** |
| --- | --- | --- | --- |
| **(1)** | $prCBOi\underset{\to}{{ktr}_{1}}prCBOi+C787+C786$ | Synthesis of inhibitory TCS components | Hill function, regulated by N |
| **(2)** | $prCBOi\underset{\to}{{ki}_{1}} prCBOi\_C2$ | Inhibition of TCS promoter | Hill function, regulated by CodY2 |
| **(3)** | $prCBOi\_C2\underset{\to}{{ku}_{1}}prCBOi$ | Reactivation of inhibitory TCS promoter | Mass action |
| **(4)** | $prBR\underset{\to}{{ktr}_{2\_1}}pr\_BR +BotR$ | Basal synthesis of *botR* | Hill function, regulated by N |
| **(5)** | $prBR\underset{\to}{{ka}_{1}} prBR\_B$ | BotR activates its own promoter | Hill function, regulated by BotR |
| **(6)** | $prBR\_B\underset{\to}{{ku}_{2}}prBR$ | *botR* promoter deactivation | Mass action |
| **7** | $prBR\_B\underset{\to}{{ktr}_{2\_2}}prBR\_B +BotR$ | BotR directed synthesis of BotR | Hill function, regulated by N |
| **(8)** | $prBR\_B\underset{\to}{{ka}_{2}} prBR\_B$_$C2$ | CodY2 activates the *botR* promoter | Hill function, regulated by CodY2 |
| **(9)** | $prBR\_B\_C2\underset{\to}{{ku}_{3}} prBR\_B$ | *botR* promoter partial deactivation | Mass action |
| **(10)** | $prBR\_B$_$C2\underset{\to}{{ktr}_{2\_3}}prBR\_B\_ C2+BotR$ | BotR+CodY2 directed synthesis of BotR | Hill function, regulated by N |
| **(11)** | $prBA\underset{\to}{{ki}_{2}} prBA\_C1$ | Inhibition of *bont* promoter by CodY1 | Hill function, regulated by CodY1 |
| **(12)** | $prBA\_C1\underset{\to}{{ku}_{4}}prBA$ | Release of CodY1 from *bont* promoter | Mass action |
| **(13)** | $prBA\underset{\to}{{ki}_{3}} prBA\_C786$ | Inhibition of *bont* promoter by C786P | Hill function, regulated by phosphorylated C786 |
| **(14)** | $prBA\_C786\underset{\to}{{ku}_{5}} prBA$ | Release of C786P from *bont* promoter | Mass action |
| **(15)** | $prBA\_C1\underset{\to}{{ki}_{4}} prBA\_C1\_C786$ | Inhibition of *bont* promoter by CodY1 and C786P | Hill function, regulated by C786P |
| **(16)** | $prBA\_C1\_C786\underset{\to}{{ku}_{6}} prBA\_C1$ | Release of C786P from *bont* promoter | Mass action |
| **(17)** | $prBA\_C786\underset{\to}{{ki}_{5}} prBA\_C1\_C786$ | Inhibition of *bont* promoter by C786P and CodY1 | Hill function, regulated by CodY1 |
| **(18)** | $prBA\_C1\_C786\underset{\to}{{ku}_{7}} prBA\_C786$ | Release of CodY1 from *bont* promoter | Mass action |
| **(19)** | $prBA\underset{\to}{{ka}_{3}} prBA\_B$ | Activation of *bont* promoter by BotR | Hill function, regulated by BotR |
| **(20)** | $prBA\_B\underset{\to}{{ku}_{8}}prBA$ | Release of BotR from *bont* promoter | Mass action |
| **(21)** | $prBA\_B\underset{\to}{{ktr}_{3\_1}}prBA\_B+BoNT$ | BotR directed synthesis of BoNT | Hill function, regulated by N |
| **(22)** | $prBA\_B\underset{\to}{{ka}_{4}} prBA\_B\_CRR$ | Activation of *bont* promoter by CRRP | Hill function, regulated by CRRP |
| **(23)** | $prBA\_B\_CRR\underset{\to}{{ku}_{9}} prBA\_B$ | Release of CRRH from *bont* promoter | Mass action |
| **(24)** | $prBA\_B\_CRR\underset{\to}{{ktr}_{3\_2}}prBA\_B\_CRR+BoNT$ | BotR+CRRH directed synthesis of BoNT | Hill function, regulated by N |
| **(25)** | $prBA\_B\underset{\to}{{ka}_{5}} prBA\_B\_C607$ | Activation of BotR bound *bont* promoter by C607P | Hill function, regulated by C607P |
| **(26)** | $prBA\_B\_C607\underset{\to}{{ku}_{10}} prBA\_B$ | Release of C607P from BotR bound *bont* promoter | Mass action |
| **(27)** | $prBA\_B\_C607\underset{\to}{{ktr}_{3\_3}}prBA\_B\_C607+BoNT$ | BotR+C607P directed synthesis of BoNT | Hill function, regulated by N |
| **(28)** | $prBA\_B\_CRR\underset{\to}{{ka}_{6}} prBA\_B\_CRR\_C607$ | Activation of BotR and CRRP bound *bont* promoter by C607P | Hill function, regulated by C607P |
| **(29)** | $prBA\_B\_CRR\_C607\underset{\to}{{ku}_{11}} prBA\_B\_CRR$ | Release of C607P from BotR and CRRP bound *bont* promoter | Mass action |
| **(30)** | $prBA\_B\_CRR\_C607\underset{\to}{{ktr}_{3\_3}}prBA\_B\_CRR\_C607+BoNT$ | BotR+CRRP+C607P directed synthesis of BoNT | Hill function, regulated by N |
| **(31)** | $prBA\_B\_C607\underset{\to}{{ka}_{6}}prBA\_B\_CRR\_C607$ | Activation of BotR and C607P bound *bont* promoter by CRRP | Hill function, regulated by CRRH |
| **(32)** | $prBA\_B\_CRR\_C607\underset{\to}{{ku}_{12}}prBA\_B\_C607$ | Release of CRRP from BotR and C607H bound *bont* promoter | Mass action |
| **(33)** | $CodY2\underset{\to}{{kf}_{1}}CodY1$ | Deactivation of CodY | Hill kinetics, regulated by N |
| **(34)** | $CodY1\underset{\to}{{kf}_{2}}CodY2$ | Activation of CodY | Mass action |
| **(35)** | $C787\underset{\to}{{kph}_{1}}C787P$ | Phosphorylation of C787 | Hill kinetics, regulated by N |
| **(36)** | $C787P+C786 \underset{\to}{{kpht}_{1}}C787+ C786P$ | Transphosphorylation of C786 | Mass action |
| **(37)** | $C786P \underset{\to}{{kdph}_{1}}CBO\_0786$ | Dephosphorylation of C786 | Mass action |
| **(38)** | $CSHK\underset{\to}{{kph}_{2}}CSHKP$ | Phosphorylation of CSHK | Hill kinetics, regulated by S |
| **(39)** | $CSHKP+CRR\underset{\to}{{kpht}_{2}}CSHK+CRRP$ | Transphosphorylation of CRR | Mass action |
| **(40)** | $CRRP \underset{\to}{{kdph}_{2}}CRR$ | Dephosphorylation of CRR | Mass action |
| **(41)** | $C608\underset{\to}{{kph}_{3}}C608P$ | Phosphorylation of C608 | Hill kinetics, regulated by N |
| **(42)** | $C608P+C607\underset{\to}{{kpht}_{3}}C608+C607P$ | Transphosphorylation of C607 | Mass action |
| **(43)** | $C607P \underset{\to}{{kdph}_{3}}C607$ | Dephosphorylation of C607 | Mass action |
| **(44)** | $C787\underset{\to}{{kdeg}_{1}}\emptyset$ | Degradation of C787 | Mass action |
| **(45)** | $C787P\underset{\to}{{kdeg}_{2}}\emptyset$ | Degradation of C787PH | Mass action |
| **(46)** | $C786\underset{\to}{{kdeg}_{3}}\emptyset$ | Degradation of C786 | Mass action |
| **(47)** | $C786P\underset{\to}{{kdeg}_{4}}\emptyset$ | Degradation of C786PH | Mass action |
| **(48)** | $BotR\underset{\to}{{kdeg}_{5}}\emptyset$ | Degradation of BotR | Mass action |
| **(49)** | $BoNT\underset{\to}{{kdeg}_{6}}\emptyset$ | Degradation of BoNT | Mass action |
