# Supplementary material for: An Integrative Approach to Computational Modelling of the Gene Regulatory Network Controlling Clostridium botulinum Type A1 Toxin Production
Source: PLoS Comput Biol. 2016 Nov 17;12(11):e1005205. doi: 10.1371/journal.pcbi.1005205 (PMC5113860; doi:10.1371/journal.pcbi.1005205)
Supplement: S2 Text — (DOCX) [file pcbi.1005205.s002.docx]

Supporting Information File 2 – Parameters and kinetic rates of sub-models

Initial state of the population sub-model: only variables having a non-zero initial states are reported.

| N | 2.2∙10^8^ (A.U.) |
| --- | --- |
| AC | 2100 (cfu/ml) |

Initial values of the variables for the gene expression sub-model

| CodY1 | 0.95 (A.U) |
| --- | --- |
| CodY2 | 0.05 (A.U.) |
| C608 | 1 (A.U.) |
| C607 | 1 (A.U.) |
| CSHK | 1 (A.U.) |
| CRR | 1 (A.U.) |
| prCBOi | 1 (A.U.) |
| prBR | 1 (A.U.) |
| prBA | 1 (A.U.) |

Kinetic rates of the model. The following definitions are used to simplify the mathematical expressions:

- $Nn$ is the normalized variable amount of nutrients, defined as $Nn=N/$2.2e+08
- $Hill(n,K,REG)$is a positive Hill function that expresses the dependence of a rate from a regulatory species ($REG$), and it is defined as follows:

$$Hill(n,K,REG)={{REG}^{n}}/\left( {REG}^{n}+K^{n} \right)$$

- $NegHill(n,K,REG)$is a negative Hill function that expresses the dependence of a rate from a regulatory species ($REG$), and it is defined as follows:

$$NegHill(n,K,REG)={K^{n}}/\left( {REG}^{n}+K^{n} \right)$$

- $Hill_{S}$is a Hill function that expresses the dependence of all the synthesis rates for the gene expression sub-model from the amount of nutrients, defined as follows:

$$Hill_{S}={\left( Nn-0.11 \right)^{2}}/\left[ \left( Nn-0.11 \right)^{2}+{0.21}^{2} \right]$$

The function $Hill_{S}$stipulates a monotonic decrease of the synthesis rate as $Nn$, with a switch point at 21% of the initial amount, and a stop of the synthesis process when the amount of nutrients falls below 11% of the initial amount.

Kinetic parameters for the population sub-model reactions

| **ID** | **Kinetic rate** | **Parameters** |
| --- | --- | --- |
| **(1)** | $k\cdot AC\cdot NegHill(n,K,Nn)$ | $k =0.25, n=1, K=0.45$ |
| **(2)** | $k\cdot RC\cdot Hill(n1,K1,Nn)\cdot Hill(n2,K2,S)$ | $k = 0.9, n1=4, K1=0.6, n2=0.8, K2=2500$ |
| **(3)** | $k\cdot RC\cdot Hill(n,K,S)$ | $k =22.0, n=4, K=7500$ |
| **(4)** | $k$ | $k = 3.5\cdot{10}^{-6}$ |
| **(5)** | $k$ | $k = 10, \alpha=1.5\cdot{10}^{-5}$ |
| **(6)** | $k$ | $k = 0.22$ |

Kinetic rates for the gene regulation sub-model:

| ***ID*** | **Kinetic rate** | **Parameters** |
| --- | --- | --- |
| **(1)** | $k\cdot prCBOi\cdot Hill_{N}$ | $k=10$ |
| **(2)** | $k\cdot prCBOi\cdot Hill(n,K,CodY2)$ | $k=10, n=2, K=0.7$ |
| **(3)** | $k$ | $k=2$ |
| **(4)** | $k\cdot prBR\cdot Hill_{N}$ | $k=0.0001$ |
| **(5)** | $k\cdot prBR\cdot Hill(n,K,BotR)$ | $k=0.75, n=2, K=0.00325$ |
| **(6)** | $k$ | $k=0.5$ |
| **(7)** | $k\cdot prBR\_B\cdot Hill_{N}$ | $k=5$ |
| **(8)** | $k\cdot prBR\_B\cdot Hill(n,K,CodY2)$ | $k=1, n=2, K=0.5$ |
| **(9)** | $k$ | $k=1$ |
| **(10)** | $k\cdot prBR\_B\_C2\cdot Hill_{N}$ | $k=10$ |
| **(11)** | $k\cdot prBA\cdot Hill(n,K,CodY1)$ | $k=0.2, n=2, K=0.75$ |
| **(12)** | $k$ | $k=1$ |
| **(13)** | $k\cdot prBA\cdot Hill(n,K,C786P)$ | $k=20, n=2, K=0.5$ |
| **(14)** | $k$ | $k=0.55$ |
| **(15)** | $k\cdot prBA\_C1\cdot Hill(n,K,C786P)$ | $k=20, n=2, K=0.5$ |
| **(16)** | $k$ | $k=0.55$ |
| **(17)** | $k\cdot prBA\_C786\cdot Hill(n,K,CodY1)$ | $k=0.2, n=2, K=0.75$ |
| **(18)** | $k$ | $k=1$ |
| **(19)** | $k\cdot prBA\cdot Hill(n,K,BotR)$ | $k=0.5, n=2, K=0.075$ |
| **(20)** | $k$ | $k=1$ |
| **(21)** | $k\cdot RC\cdot SC\cdot prBA\_B\cdot Hill_{N}$ | $k=0.00025$ |
| **(22)** | $k\cdot prBA\_B\cdot Hill(n,K,CRRP)$ | $k=2, n=2, K=0.75$ |
| **(23)** | $k$ | $k=0.5$ |
| **(24)** | $k\cdot prBA\_B\_CRR\cdot RC\cdot SC\cdot Hill_{N}$ | $k=0.00025$ |
| **(25)** | $k\cdot prBA\_B\cdot Hill(n,K,C607P)$ | $k=2, n=2, K=1$ |
| **(26)** | $k$ | $k=0.5$ |
| **(27)** | $k\cdot prBA\_B\_C607\cdot RC\cdot SC\cdot Hill_{N}$ | $k=0.00025$ |
| **(28)** | $k\cdot prBA\_B\_CRR\cdot Hill(n,K,C607P)$ | $k=2, n=2, K=1$ |
| **(29)** | $k$ | $k=0.5$ |
| **(30)** | $k\cdot prBA\_B\_CRR\_C607\cdot RC\cdot SC\cdot Hill_{N}$ | $k=0.0004$ |
| **(31)** | $k\cdot prBA\_B\_C607\cdot Hill(n,K,CRRP)$ | $k=2, n=2, K=1$ |
| **(32)** | $k$ | $k=0.5$ |
| **(33)** | $k$ | $k=10$ |
| **(34)** | $k\cdot CodY1\cdot NegHill(n,K,N_{n})$ | $k=100, n=20, K=0.8$ |
| **(35)** | $k\cdot C787\cdot Hill(n,K,N_{n})$ | $k=10, n=2, K=0.75$ |
| **(36)** | $k$ | $k=10$ |
| **(37)** | $k$ | $k=0.1$ |
| **(38)** | $k\cdot CSHK\cdot Hill(n,K,S)$ | $k=0.5, n=2, K=500$ |
| **(39)** | $k$ | $k=10$ |
| **(40)** | $k$ | $k=1$ |
| **(41)** | $k\cdot C608\cdot NegHill(n,K,N_{n})$ | $k=5, n=2, K=0.81$ |
| **(42)** | $k$ | $k=10$ |
| **(43)** | $k$ | $k=1$ |
| **(44)** | $k$ | $k=10$ |
| **(45)** | $k$ | $k=10$ |
| **(46)** | $k$ | $k=10$ |
| **(47)** | $k$ | $k=10$ |
| **(48)** | $k$ | $k=10$ |
| **(49)** | $k$ | $k=0.275$ |
