# Supplementary material for: An Integrative Approach to Computational Modelling of the Gene Regulatory Network Controlling Clostridium botulinum Type A1 Toxin Production
Source: PLoS Comput Biol. 2016 Nov 17;12(11):e1005205. doi: 10.1371/journal.pcbi.1005205 (PMC5113860; doi:10.1371/journal.pcbi.1005205)
Supplement: S3 Text — (DOCX) [file pcbi.1005205.s003.docx]

Supporting Information File 3 – Details of the sequencing study

Experimental studies on other bacteria including *Bacillus subtilis* and *Lactococcus lactis*, using DNase I footprinting and mutational analysis have shown that a DNA sequence motif is associated with CodY binding [[1-3](#_ENREF_1)]. This has most often been characterised by the palindromic consensus motif of 15 bp AATTTTCWGAAAATT, sometimes specified as AATTTTCWGAAAATT or the close variant AATTTTGNCAAAATT [[2](#_ENREF_2)]. Many instances of the binding motif supported by experimental evidence differ from the consensus by 3 or 4 mismatch positions, with some differing by as many as 5 mismatches [[2](#_ENREF_2),[4](#_ENREF_4)]. More recently this has been characterised in the form of longer variant motifs/sequence logos of 17 [[5](#_ENREF_5)] and 24 bp [[6](#_ENREF_6)]. The latter in essence is equivalent to two overlapping instances of the traditional consensus. In *C. botulinum* ATCC3502, two instances of the 15-bp motif, differing from the consensus by 3 mismatches each, have been previously identified in the intergenic region upstream of a CodY-regulated gene (*ntnh*), close to the core promoter [[7](#_ENREF_7)]. One of these occurs in a 30-bp region which was shown to be protected by CodY in DNase I footprinting.

We examined the 226-bp intergenic region upstream of *botR* in *C. botulinum* strain ATCC 3502 (NCBI RefSeq NC_009495.1 bp 905,160-905,385). The following coordinates refer to bp 1 corresponding to bp 905,160 of the full chromosome sequence, and lower-case letters indicate mismatches to the specified motif. A 15-bp sequence AATTTaaAGgcAATT, differing from the consensus by 4 mismatches, occurs at positions 153-167, although this lacks the strongly conserved A at position 10. Perhaps more interestingly, this overlaps with one of three closely spaced instances (104-118, 128-142, 143-157) of one of the *C. botulinum* motifs associated with the CodY-binding region of the *ntnh-bont* promoter [[7](#_ENREF_7)] - each of these mismatches at 3 or 4 positions relative to that 15-bp sequence. Moreover, two of these lie partially or wholly within a 32-bp motif which matches 20 of the 30 bp of the 30-bp protected segment (positions 112-143, including a single 2-bp insertion). This 32-bp sequence, with the insertion in italic, is cATAAtTGATtA*TG*GAtatTtCgtAAAAATGg. The 15-bp motif it wholly includes near its 3' end differs from the AATTTTGNCAAAATT consensus [[2](#_ENREF_2)] at 4 positions (tATTTcGTaAAAATg).

Until further experiments are performed, it remains hypothetical whether *botR* is regulated by CodY binding to these motifs; but we note the above similarities to the CodY-binding region in the upstream region of *ntnh-bont* operon, which is consistent with a similar mode of binding of *botR.*

1. den Hengst CD, van Hijum SAFT, Geurts JMW, Nauta A, Kok J, et al. (2005) The Lactococcus lactis CodY Regulon: IDENTIFICATION OF A CONSERVED cis-REGULATORY ELEMENT. Journal of Biological Chemistry 280: 34332-34342.

2. Belitsky BR, Sonenshein AL (2008) Genetic and Biochemical Analysis of CodY-Binding Sites in Bacillus subtilis. Journal of bacteriology 190: 1224-1236.

3. Guédon E, Sperandio B, Pons N, Ehrlich SD, Renault P (2005) Overall control of nitrogen metabolism in Lactococcus lactis by CodY, and possible models for CodY regulation in Firmicutes. Microbiology 151: 3895-3909.

4. Barbieri G, Albertini AM, Ferrari E, Sonenshein AL, Belitsky BR (2016) Interplay of CodY and ScoC in the Regulation of Major Extracellular Protease Genes of Bacillus subtilis. Journal of Bacteriology 198: 907-920.

5. Belitsky BR, Sonenshein AL (2013) Genome-wide identification of Bacillus subtilis CodY-binding sites at single-nucleotide resolution. Proceedings of the National Academy of Sciences 110: 7026-7031.

6. Wray LV, Fisher SH (2011) Bacillus subtilis CodY Operators Contain Overlapping CodY Binding Sites. Journal of bacteriology 193: 4841-4848.

7. Zhang Z, Dahlsten E, Korkeala H, Lindström M (2014) Positive Regulation of Botulinum Neurotoxin Gene Expression by CodY in Clostridium botulinum ATCC 3502. Applied and Environmental Microbiology 80: 7651-7658.
